# Wnt signaling during tooth replacement in zebrafish (*Danio rerio*): pitfalls and perspectives

**DOI:** 10.3389/fphys.2014.00386

**Published:** 2014-10-06

**Authors:** Ann Huysseune, Mieke Soenens, Fien Elderweirdt

**Affiliations:** Evolutionary Developmental Biology Research Group, Biology Department, Ghent UniversityGhent, Belgium

**Keywords:** Wnt, tooth replacement, zebrafish, dickkopf, polyphyodont, axin, APC, LiCl

## Abstract

The canonical (β-catenin dependent) Wnt signaling pathway has emerged as a likely candidate for regulating tooth replacement in continuously renewing dentitions. So far, the involvement of canonical Wnt signaling has been experimentally demonstrated predominantly in amniotes. These studies tend to show stimulation of tooth formation by activation of the Wnt pathway, and inhibition of tooth formation when blocking the pathway. Here, we report a strong and dynamic expression of the soluble Wnt inhibitor *dickkopf1* (*dkk1*) in developing zebrafish (*Danio rerio*) tooth germs, suggesting an active repression of Wnt signaling during morphogenesis and cytodifferentiation of a tooth, and derepression of Wnt signaling during start of replacement tooth formation. To further analyse the role of Wnt signaling, we used different gain-of-function approaches. These yielded disjunct results, yet none of them indicating enhanced tooth replacement. Thus, masterblind (*mbl*) mutants, defective in *axin1*, mimic overexpression of Wnt, but display a normally patterned dentition in which teeth are replaced at the appropriate times and positions. Activating the pathway with LiCl had variable outcomes, either resulting in the absence, or the delayed formation, of first-generation teeth, or yielding a regular dentition with normal replacement, but no supernumerary teeth or accelerated tooth replacement. The failure so far to influence tooth replacement in the zebrafish by perturbing Wnt signaling is discussed in the light of (i) potential technical pitfalls related to dose- or time-dependency, (ii) the complexity of the canonical Wnt pathway, and (iii) species-specific differences in the nature and activity of pathway components. Finally, we emphasize the importance of in-depth knowledge of the wild-type pattern for reliable interpretations. It is hoped that our analysis can be inspiring to critically assess and elucidate the role of Wnt signaling in tooth development in polyphyodonts.

## Introduction

**Figure 1:**
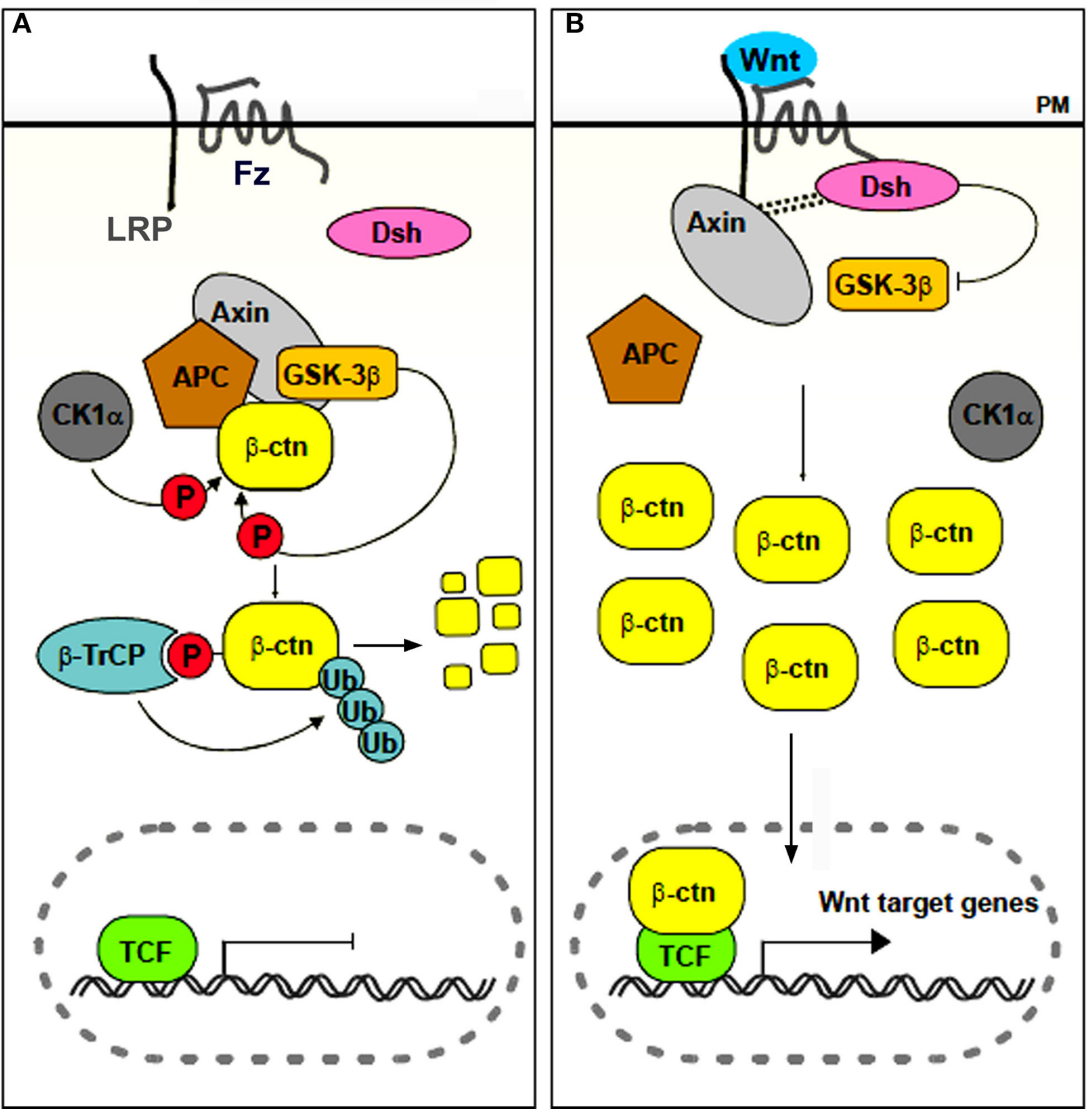
**Simplified scheme of canonical Wnt signaling**. In the absence of a ligand **(A)**, or in the presence of an inhibitor, β-catenin accumulates in the cytoplasm and is targeted for destruction. In the presence of a ligand **(B)**, the destruction complex is disassembled, β-catenin accumulates and translocates to the nucleus, where it associates with transcription factors of the LEF/TCF family to regulate transcription of target genes. Abbreviations: APC, Adenomatous Polyposis Coli; β-ctn, β-catenin; β-TrCP, β-transducin-repeat-containing protein; CK1α, casein kinase 1α; Dsh, Disheveled; Fz, frizzled; GSK-3β, glycogen synthase kinase 3β; LRP, low density lipoprotein receptor related protein; P, phosphorylated; PM, plasma membrane; TCF, T Cell Factor; Ub, Ubiquitin. Adapted from Denayer (2006).

Chondrichthyans, actinopterygians (amongst which the vast group of teleosts) and non-mammalian sarcopterygians (except for some edentulous taxa such as turtles and birds) typically renew their teeth throughout life (so-called polyphyodont dentition) (Huysseune and Sire, 1998; Huysseune et al., 2005). Recently, the cellular and molecular mechanisms responsible for this capacity have become the focus of an exploding field of research (reviewed in Richman and Handrigan, 2011; Jernvall and Thesleff, 2012; Tucker and Fraser, 2014). Apart from exploring the potential involvement of stem cells, the focus lies on the different signaling pathways that may be involved in tooth replacement. One, in particular, has emerged as a likely candidate for controling tooth replacement in polyphyodont animals: the canonical, or β-catenin dependent, Wnt signaling pathway (Huysseune and Thesleff, 2004; Handrigan and Richman, 2010; Gaete and Tucker, 2013; Weeks et al., 2013). Briefly, this pathway is activated when soluble glycoproteins of the Wnt family bind as a ligand to a Frizzled (Fz) receptor and low density lipoprotein receptor-related protein 5/6 (LRP5/6) co-receptor. In the absence of the ligand, a multiprotein complex that includes Adenomatous Polyposis Coli (APC), axin, casein kinase 1α (CK1α) and glycogen synthase kinase-3β (GSK-3β) mediates the destruction of cytoplasmically localized β-catenin. Upon binding of the ligand, the destruction complex becomes disassembled, and β-catenin translocates to the nucleus, where it associates to transcription factors of the LEF-1/TCF family and promotes (in some cases inhibits) transcription of Wnt-responsive target genes (for reviews see Logan and Nusse, 2004; Clevers and Nusse, 2012; Fagotto, 2013; Gao et al., 2014; Song et al., 2014, and the Wnt homepage, http://wnt.stanford.edu) (Figure 1). The Wnt signaling pathway can be inhibited in various ways. For example Dickkopf1, DKK1, is a soluble inhibitor that blocks the LRP5/6 receptor thus preventing ligands to bind (Glinka et al., 1998; Niehrs, 2006; Bao et al., 2012). At the same time, *dkk1* is also a target gene of the Wnt signaling pathway, thus acting in a negative feedback loop (Niida et al., 2004).

So far, functional analyses demonstrating the involvement of the Wnt pathway in tooth replacement have been essentially limited to amniotes. Wnt gain-of-function in mammals usually leads to enhanced tooth development and/or supernumerary teeth. Thus, in humans, loss of *APC*, and therefore inactivation of the β-catenin destruction complex, frequently leads to hyperdontia (Thakker et al., 1995). Paradoxically, loss of *AXIN2*, which codes for a protein belonging to the same destruction complex, has an opposite effect, and leads to severe hypodontia (Lammi et al., 2004). Tooth germs in mice expressing stabilized β-catenin in the epithelium (i.e., mimicking continuous Wnt signaling) give rise to multiple teeth (Järvinen et al., 2006) or the production of multiple tooth-like epithelial protrusions (Liu et al., 2008); constitutive stabilization of β-catenin in the developing palatal mesenchyme induces aberrant palatal epithelial invaginations resembling early tooth buds (Chen et al., 2009). Mice deficient in *Apc* produce supernumerary teeth (Wang et al., 2009; Wang and Fan, 2011). Mice deficient in *Lrp4*, a co-receptor that inhibits Wnt signaling, show supernumerary tooth formation in the diastema that is accompanied by upregulation of canonical Wnt signaling (Porntaveetus et al., 2012). Inactivation of *Wise* (also called Sostdc1, USAG-1, and ectodin), an inhibitor of Lrp5- and Lrp6-dependent Wnt signaling, likewise leads to elevated Wnt signaling and supernumerary teeth (Munne et al., 2009; Ahn et al., 2010). Conversely, loss-of-function experiments in mice usually lead to disturbed odontogenesis. Thus, ectopic expression of the Wnt antagonist Dickkopf1 (*Dkk1*) arrests tooth morphogenesis at the early bud stage (Andl et al., 2002); ectopic Wise reduces Wnt signaling and tooth number (Ahn et al., 2010). Ectopic application of Mfrzb1 protein, another Wnt antagonist, leads to retarded tooth development and the formation of smaller teeth (Sarkar and Sharpe, 2000). Inactivation of Gpr177 (whose product regulates Wnt sorting and secretion) in dental epithelium leads to inhibition of Wnt signaling activity and arrest of early tooth development (Zhu et al., 2013). Inactivation of β-catenin in the developing tooth mesenchyme causes developmental arrest at the bud stage (Chen et al., 2009). Overall, inhibition of Wnt signaling activity in mice, either in the dental epithelium or in the mesenchyme leads to arrest of early tooth development (reviewed by Lan et al., 2014). Turning to squamates, in the gecko *Eublepharis macularius*, Wnt gain-of-function induces proliferation of the dental lamina (Handrigan et al., 2010), while in the python *Python regius* canonical Wnt signaling promotes proliferation in dental explants (Handrigan and Richman, 2010). In the corn snake (*Pantherophis guttatus*), activation of the Wnt/β-catenin pathway in culture increases the number of developing tooth germs (Gaete and Tucker, 2013). Stimulation of Wnt signaling *in vitro* in the American alligator (*Alligator mississippiensis*) induces dental lamina proliferation and distal expansion, mimicking changes observed during normal initiation of tooth formation, whereas Wnt inhibition can block the growth of replacement teeth (Wu et al., 2013). Taken together, these studies in amniotes tend to show stimulation of tooth formation by activation of the Wnt pathway, and inhibition of tooth formation when blocking the pathway. Very few studies have addressed the role of Wnt signaling in actinopterygian tooth formation so far. Parsons et al. (2014) report accelerated development of teeth in zebrafish after activating the Wnt pathway with LiCl. In contrast, Fraser et al. (2013) found that LiCl appears to delay tooth replacement in two species of cichlids.

**Figure 2:**
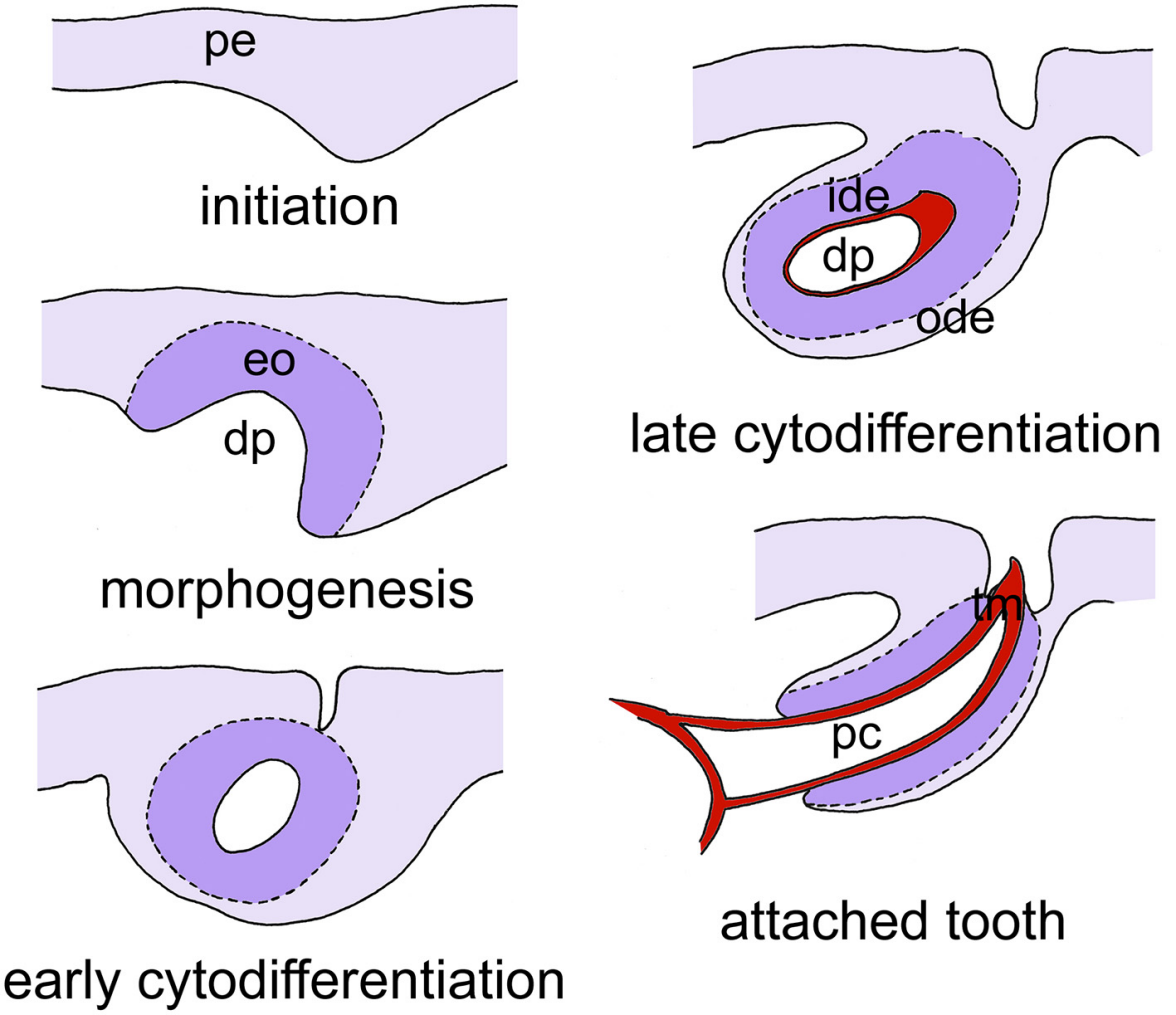
**Simplified scheme of tooth development in zebrafish, as exemplified for tooth 4V^1^**. Tooth development proceeds through five successive, and partially overlapping stages, initiation, morphogenesis, early cytodifferentiation, late cytodifferentiation, and attachment. Only the epithelium is color-coded. Abbreviations: dp, dental papilla; eo, enamel organ; ide, inner dental epithelium; ode, outer dental epithelium; pc, pulp cavity; pe, pharyngeal epithelium; tm, tooth matrix.

The obvious question therefore remains what the role is of Wnt signaling in the control of tooth replacement in actinopterygians and whether its implication could represent an ancient mechanism rooted deep in osteichthyan (and perhaps even gnathostome) phylogeny. To address this question, we have embarked on studies in the zebrafish (*Danio rerio*). This cyprinid has pharyngeal teeth only that are located in three rows on each of the fifth ceratobranchials. They develop in a stereotypical pattern, which has been well-characterized (Van der heyden and Huysseune, 2000). At a standard temperature of 28.5°C, the first tooth germ appears in the fourth position of the ventral (V) tooth row, and is therefore called 4V^1^, the superscript indicating the generation number (Figure 2). This tooth starts to develop at 48 h post-fertilization (hpf) and is attached by 80 hpf. The next two germs arise in the third and fifth position in the ventral tooth row (3V^1^ and 5V^1^) and start to develop at 56hpf. The first replacement tooth germ, at position 4V (4V^2^), already starts to form at 80 hpf (Borday-Birraux et al., 2006) (Table 1).

**Table 1:**
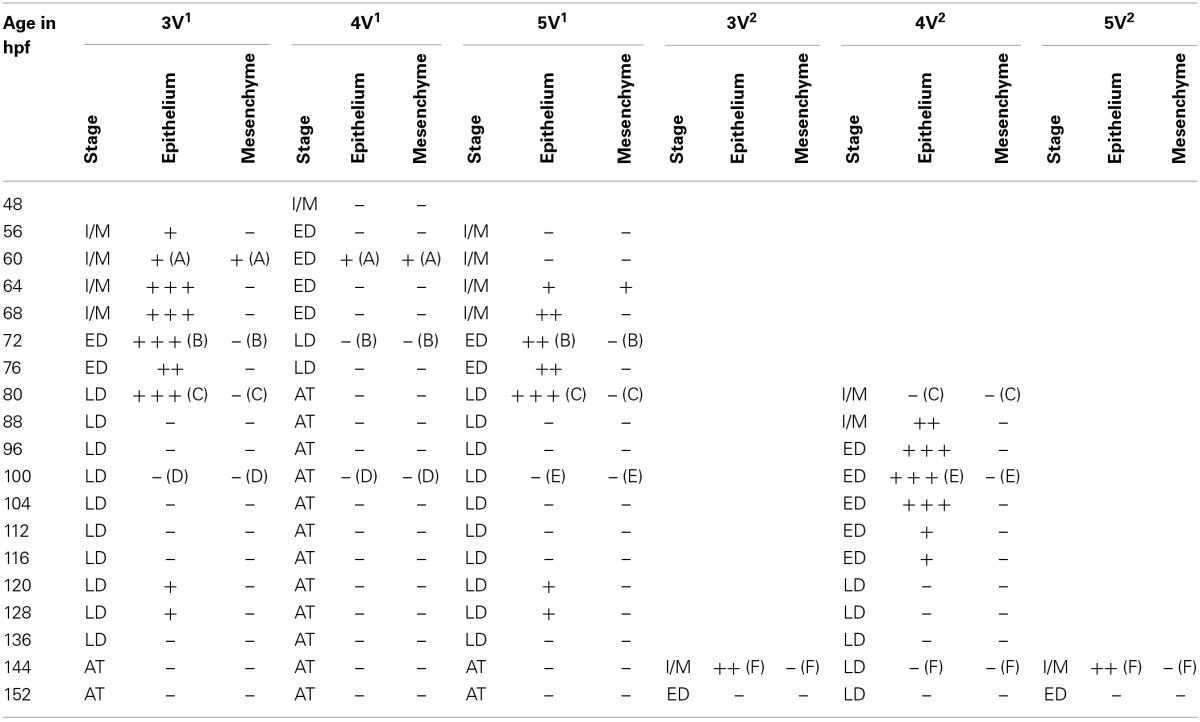
**Expression of *dkk1* in epithelium and mesenchyme of first-generation teeth (3V^1^, 4V^1^, and 5V^1^) and their successors (3V^2^, 4V^2^, and 5V^2^) between 48 and 152 hpf**.

**Figure 3:**
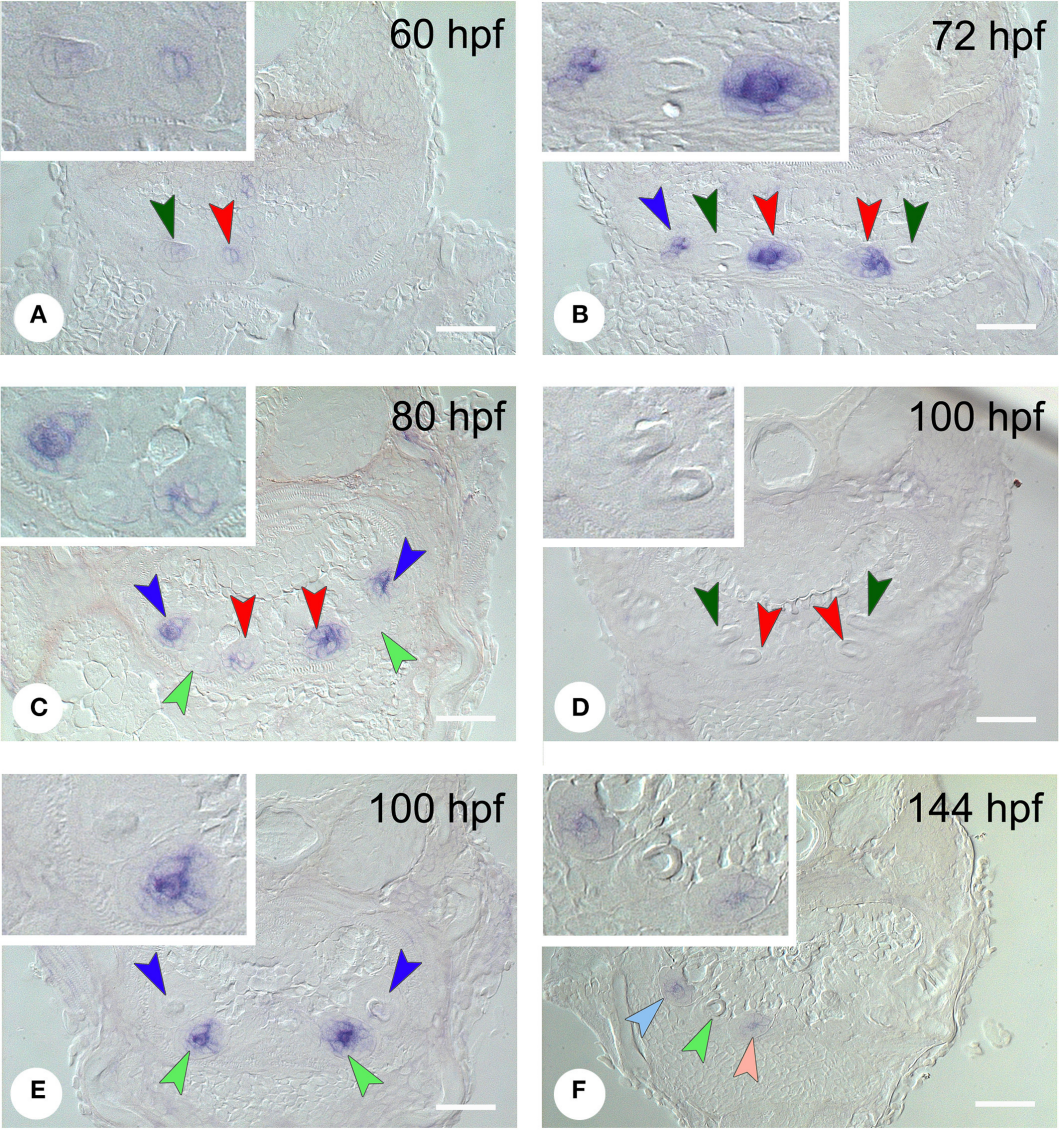
**Expression of *dkk1* in different stages of development of first- and second-generation teeth**. **(A)** 60 hpf: weak mesenchymal expression in tooth 4V^1^, epithelial expression starts to be upregulated in 3V^1^; **(B)** 72 hpf: strong epithelial expression in 3V^1^ and 5V^1^, but loss of signal in 4V^1^; **(C)** 80 hpf: start of formation of 4V^2^ showing no signal yet; epithelial signal is weakening in 3V^1^ and 5V^1^; **(D,E)** 100 hpf; *dkk1* is now downregulated in all first-generation teeth [3V^1^ and 4V^1^ in **(D)**, 5V^1^ in **(E)**], whilst the gene is upregulated in the first replacement tooth (4V^2^); **(F)** 144 hpf: expression in the two next second-generation teeth, 3V^2^ and 5V^2^. Dark-colored arrowheads indicate specific first-generation teeth and point downwards: dark green, 4V^1^, red, 3V^1^, dark blue, 5V^1^; light-colored arrowheads indicate second-generation teeth and point upwards: light green, 4V^2^, pink, 3V^2^, light blue, 5V^2^. Note that epithelium and mesenchyme of a tooth germ are not necessarily visible on a single section through the germ. Sections may also be slightly oblique and different sets of teeth may be visible on either body side. Insets show higher magnification of the teeth on the left side in each micrograph. Scale bars = 50 μm.

*Stages of tooth development are identified as initiation and morphogenesis (I/M), early cytodifferentiation (ED), late cytodifferentiation (LD) and attached tooth (AT), in agreement with Figure 2. Level of expression is scored from – (no expression) to +++ (very strong expression). Letters following a signal refer to **Figure 3** (A–F) where the tooth is visualized, so that a particular expression signal in the Table can be compared with a specific image in **Figure 3***.

Here, we demonstrate the dynamic expression of the soluble Wnt inhibitor *dkk1* during zebrafish tooth formation. We subsequently use gain-of-function approaches to investigate the role of Wnt signaling in tooth replacement in the zebrafish. These approaches include the study of mutants defective in proteins of the β-catenin destruction complex, and pharmacological inhibition targeting the destruction complex. Surprisingly, stimulation of the Wnt pathway tends to disturb, rather than stimulate tooth formation in this model. We have therefore engaged in a critical analysis to assess Wnt involvement in tooth replacement in this and other polyphyodont models.

## Materials and methods

### Animal husbandry and mutant lines

Mutant zebrafish defective in *axin1* (masterblind, *mbl*) (Heisenberg et al., 2001; van de Water et al., 2001) were a gift from the Hubrecht Laboratory, Utrecht, the Netherlands. A total of 11 embryos aged between 3 and 5 days post-fertilization (dpf) was processed for serial sectioning and reconstruction of the dentition (see below). Mutants defective in Adenomatous Polyposis Coli, *apc* (Hurlstone et al., 2003) were generously donated by Hans Clevers, Hubrecht Laboratory, Utrecht, the Netherlands. For both mutants, age-matched wildtype (WT) and heterozygous mutants were processed as controls.

### ISH of *dkk1*

The product of the gene *dkk1* is a soluble inhibitor of Wnt signaling. Plasmids containing the coding sequence of the *dkk1* gene (Hashimoto et al., 2000) were a generous gift of Dr. M. Hibi (RIKEN, Kobe, Japan). These were used to generate DIG-labeled antisense RNA probes for whole mount ISH in closely staged (interval of 4–8 h) embryos and larvae, starting at 48 hpf up to 152 hpf. The gene *dkk1* was later renamed *dkk1b* (Untergasser et al., 2011).

### Gain-of-function approaches

LiCl activates the Wnt pathway by inhibiting GSK-3β activity, thus preventing proper functioning of the β-catenin destruction complex, in this way mimicking continuous Wnt-signaling. We used a transient treatment with 300 mM LiCl, shown by Robertson et al. (2014) to result in a robust eyeless phenotype at 24 hpf. We applied LiCl both *in vivo* and *in vitro*, since the latter offers the possibility to maintain the tissues much longer while exposed to LiCl than would be possible with live fish. For *in vivo* treatments, we used 300 mM for 10 min. or 1 h (*N* = 34), at developmental stages varying between 45 and 112 hpf, and allowed the fish to survive up to 9 dpf. Untreated age-matched fish from the same batch were used as controls at different time points (*N* = 18). For *in vitro* culture, we followed the protocol described in Van der heyden et al. (2005). We explanted heads of 48 hpf fish, and cultured them for 4 days exposing them to LiCl with different concentrations (1, 5, 30, 300 mM) for either 1 h (followed by recovery in the regular culture medium) (*N* = 8) or continuously throughout the culture period (i.e., for 4 days) (*N* = 10). Numbers indicate explants that were successfully recovered after the culture period. Controls were incubated in the medium without LiCl, or were exposed to KCl instead of LiCl (*N* = 18).

### Further processing and analysis

Mutant and pharmacologically treated fish, as well as their controls, were sacrificed according to the Belgian law on the protection of laboratory animals (KB d.d. 13 September 2004) by an overdose of the anaesthetic MS222, fixed in paraformaldehyde-glutaraldehyde, embedded in epon, serially sectioned into 1 μm cross sections, stained with toluidine blue and mounted in DePex, as described previously (Huysseune and Sire, 1992). Specimens used for ISH were likewise sacrificed and, following ISH, embedded in epon and serially sectioned into 4 μm cross sections, as described in Verstraeten et al. (2012). Serial sections are required for detecting young tooth germs prior to any matrix deposition, as well as to identify their developmental stage and the tissue layers showing expression. Inevitably this procedure reduces the number of specimens that can be analyzed within reasonable time limits.

All sections were examined using a Zeiss Axio Imager compound microscope. Photographs were made with an Axio MRC camera. A schematic representation of the dentition of each specimen was obtained based either on superimposition of drawings made by camera lucida, or on serial photographs.

## Results

*In situ* hybridization revealed the spatiotemporal expression domain of *dkk1*, a strong soluble inhibitor of Wnt signaling. The gene is strongly expressed in the epithelium of the first-generation teeth that develop during the observed time interval, as well as of their successors (Table 1 and Figure 3, *N* = 22). Transcripts are first detected at 56 hpf in tooth 3V^1^. A faint and transient mesenchymal expression is observed at 60-64 hpf in each of the three first-generation teeth, but otherwise, the expression is exclusively epithelial. Epithelial expression starts to be upregulated at 60 hpf in tooth 3V^1^ (Figure 3A) and at 68 hpf in tooth 5V^1^. At 72 hpf, for example, strong epithelial expression can be observed in both these teeth (Figure 3B). At 80 hpf, replacement tooth 4V^2^ starts to form but does not show any signal yet (Figure 3C). The epithelial signal is now weakening in 3V^1^ and 5V^1^ and is lost altogether at 88 hpf. While, at 100 hpf, all first-generation teeth have lost *dkk1* expression (Figures 3D,E), the gene is now clearly upregulated in the first replacement tooth (4V^2^) (Figure 3E). Likewise, once the replacement teeth have been initiated in positions 3V and 5V (i.e., teeth 3V^2^ and 5V^2^), they display epithelial expression (Figure 3F). In summary, the gene is expressed from morphogenesis stage onwards, and is downregulated in late cytodifferentiation stage both in first-generation teeth and their immediate successors. The gene is only transiently, and very faintly, expressed in the mesenchyme of the first-generation teeth, but not in the replacement teeth. Remarkably, the expression is much weaker in the very first tooth to form, 4V^1^, where it disappears precociously at 64 hpf.

**Figure 4:**
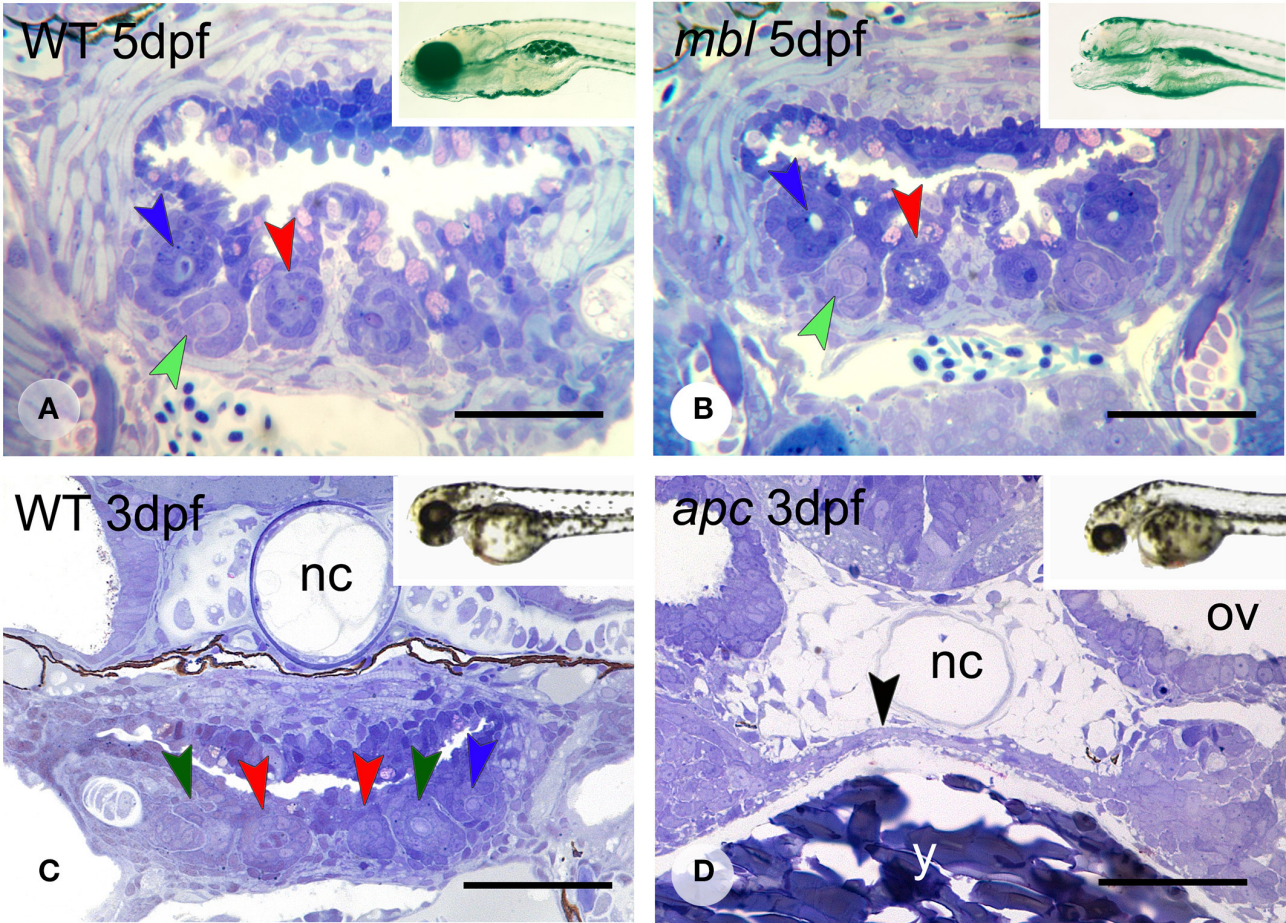
**Dentition in *mbl* and *apc* mutant zebrafish**. Dentition in WT **(A)** and *mbl* mutants **(B)** at 5dpf, and WT **(C)** and *apc* mutants **(D)** at 3 dpf. Insets: WT **(A)** and *mbl* mutant **(B)** at 6 dpf, and WT **(C)** and *apc* mutant **(D)** at 2 dpf. Color codes for arrowheads as in Figure 3. Note the complete similarity in tooth pattern between 5 dpf WT **(A)** and *mbl* mutants **(B)**, and the substantial developmental delay in 3 dpf *apc* mutants **(D)** compared to WT fish **(C)**, as can be observed from the large amount of yolk (y) still present, the thin endodermal layer covering the yolk (black arrowhead), and the absence of cartilage in the neurocranial base flanking the notochord (nc). Ov, otic vesicle. Note that sections are slightly oblique and different sets of teeth are visible on either body side. Scale bars = 50 μm.

Masterblind (*mbl*, *axin1*) mutants, which carry a point mutation in the GSK-3β-binding domain (Heisenberg et al., 2001; van de Water et al., 2001) are early lethal and die by about 6 dpf. They exhibit a dramatic phenotype with forebrain and eyes missing (Figures 4A,B, insets), as has been reported before (Heisenberg et al., 2001; van de Water et al., 2001). Remarkably, they possess a normally patterned dentition, displaying three first-generation teeth at 3 days (*N* = 5) in developmental stages that match those in the WT fish (*N* = 2) (data not shown). Likewise, at 4 and 5 dpf (*N* = 6), they possess three first-generation teeth and one replacement tooth, again in developmental stages that match those in the WT fish (*N* = 2) (Figures 4A,B). In two specimens at 5 dpf, the replacement tooth was still in morphogenesis, rather than cytodifferentiation stage.

Like *mbl* mutants, *apc* mutants—caused by a premature stop codon—(Hurlstone et al., 2003), are early lethal. However, they barely survive beyond 3 dpf. At this age, the mutants display an external phenotype with a deflected head and a large yolk sac suggestive of a developmental delay (Figures 4C,D, insets). This delay was confirmed in serial sections (*N* = 3): the pharyngeal region in 3 dpf mutants is poorly differentiated with at most two layers of flattened endodermal cells surmounting a large yolk sac (Figure 4D) and immediately adjacent to the notochord. There is no pharyngeal cavity yet. Mesenchymal cells are largely confined to the forming pharyngeal arches more laterally but absent in the region where the teeth will form. In accordance with this immature state, there is no evidence of any tooth germ in these mutants, in contrast to 3 dpf WT fish, which display three tooth germs at this time point (Figure 4C).

**Figure 5:**
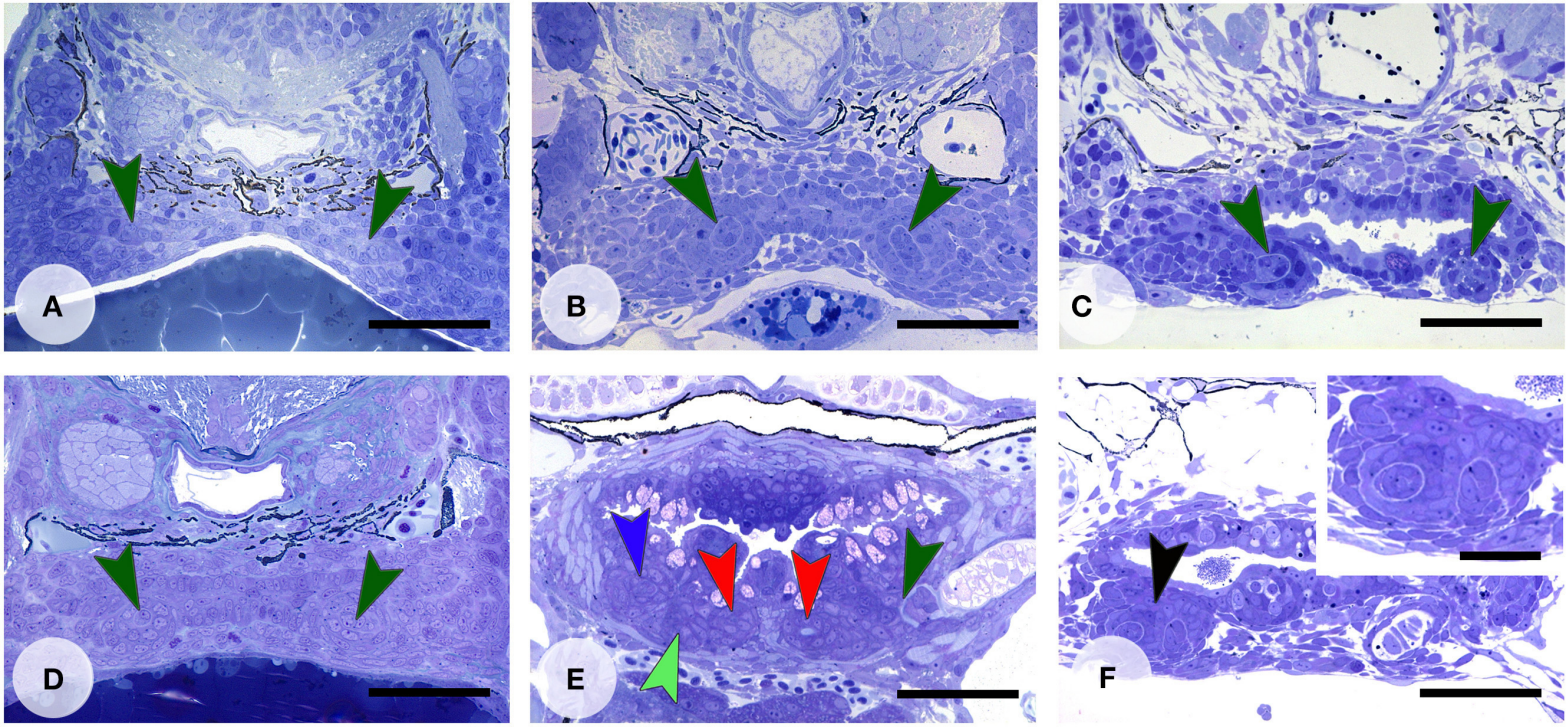
**Enhanced Wnt signaling through LiCl treatment**. Dentition of control zebrafish at start of treatment at 45 hpf **(A)**, and treated with 300 mM LiCl for 1 h (**B**, fixed at 60 hpf and **C**, fixed at 112 hpf). In **(B,C)** only tooth 4V^1^ is present. Dentition of control zebrafish at start of treatment at 72 hpf **(D)**, and treated with 300 mM LiCl for 1 h (**E**, fixed at 92 hpf), or treated with 300 mM LiCl for 10 min (**F**, fixed at 112 hpf). **(E)** All three first-generation teeth (3V^1^, 4V^1^, and 5V^1^) have formed and replacement tooth 4V^2^ is in morphogenesis stage (note that the section is slightly oblique and different sets of teeth are visible on either body side); **(F)** several tooth buds appear to form from a single enamel organ (black arrowhead, inset). Color codes for arrowheads as in Figure 3. Scale bars = 50 μm for **A–F** and 20 μm for inset.

Activation of the Wnt pathway was attempted in various ways. LiCl was applied *in vivo* before the start of formation of the first tooth in the dentition, 4V^1^ (Figure 5A). After a longer exposure time (1 h), applied at around 45 hpf, the first tooth (4V^1^) was formed, but not the flanking teeth 3V^1^ and 5V^1^ (Figures 5B,C) (*N* = 4/4), although the defect was restored afterwards, and the dentition became indistinguishable from controls at 9 dpf (data not shown) (*N* = 3/3). A later start of the treatment (at 72 hpf, Figure 5D) had variable effects, not consistent with the length of the treatment, yielding either a normal dentition (*N* = 3/5), or a reduced number of primary teeth (*N* = 2/5). A shorter exposure time (10 min.) yielded a normally patterned dentition (*N* = 20/22) including the presence of replacement teeth (Figure 5E), albeit often (*N* = 11/20) with a slight delay in the developmental stage of the replacement teeth compared to age-matched controls. In two specimens, treated for 10 min, (*N* = 2/22) several buds could be observed issuing from an unusual folding of an apparently single enamel organ (Figure 5F). This finding could, however, not be reproduced. In summary, activating the pathway with LiCl had variable outcomes, either resulting in the absence, or the delayed formation, of first-generation teeth, or yielding a regular dentition with normal replacement, but no supernumerary teeth or accelerated tooth replacement.

Culture of head explants for 4 days in the presence of LiCl 5 mM yielded 4V^1^ attached only, a result not significantly different from the (variable) results obtained either for regular medium or medium with 5 mM KCl added. When exposed for 1 h only, all three first-generation teeth tended to be present, occasionally even with the first replacement tooth, in lower concentrations of LiCl (1 or 5 mM). In higher concentrations (30 mM) the number of primary teeth was reduced to one or two, a result again not dissimilar from treatment with KCl (data not shown).

## Discussion

We report a strong and dynamic expression of the soluble Wnt inhibitor *dkk1* in tooth germs of zebrafish embryos between 48 and 152 hpf. Dickkopf expression in rodents (mouse, rat) has been reported mostly for late stages of tooth development (odontoblast differentiation, Fjeld et al., 2005; Moriguchi et al., 2013). In the mouse mandibular molar, expression of *Dkk1* shifts from the epithelium at bud stage to the mesenchyme during further odontogenesis (Monaghan et al., 1999; Fjeld et al., 2005), a pattern that is distinct from the exclusive epithelial expression reported here. Our results may be more consistent with the expression in the foregut endoderm reported by Glinka et al. (1998) in the mouse and the predominantly epithelial (epidermal) expression reported by Untergasser et al. (2011) in the zebrafish. Given the role of *dkk1* as an inhibitor of Wnt signaling, the spatiotemporal pattern of expression of *dkk1* reported here suggests an active repression of Wnt signaling during the phases of morphogenesis and cytodifferentiation of the first-generation teeth, as well as of their successor. Replacement tooth formation starts after late cytodifferentiation, more precisely following attachment and eruption of the predecessor (Huysseune, 2006). Thus, tooth replacement starts in a time window between downregulation of *dkk1* expression in the predecessor, and upregulation in its successor. If we consider expression to be a proxy for an active protein, it can be presumed that downregulation of *dkk1* at late cytodifferentiation could remove the inhibition, allowing Wnt signaling to occur. Repression and derepression as inferred from expression of *dkk1* of course assumes constant levels of Wnt ligand, so that increased levels of expression of the inhibitor are adequate to change levels of signal transduction. This assumption may be tested by using other readouts of Wnt signaling, such as *axin2* (discussed below). The dynamic pattern of *dkk1* expression during tooth replacement resembles the pattern observed during budding of neuromasts from existing neuromasts in zebrafish, although in the latter case, the dickkopf gene involved is *dkk2* (Wada et al., 2013). Since *dkk1* is itself a target gene of Wnt signaling (Niida et al., 2004), upregulation of *dkk1* once the replacement tooth is in morphogenesis stage could be the result of a negative feedback loop.

Given the strong and dynamic *dkk1* expression, we have used various gain-of-function approaches to test the role of Wnt signaling in tooth development and replacement, all of which work by interfering with the β-catenin destruction complex. If developmental processes are conserved between actinopterygians and sarcopterygians, one could expect that stimulating the Wnt pathway would lead to a hyperdontic condition, with supernumerary teeth and/or accelerated replacement. Conversely, along the same lines, interfering with the signaling pathway should result in disrupted tooth formation and/or replacement and a hypodontic condition. Published results on Wnt loss-of-function in zebrafish at least seem to partially support this hypothesis: a *lef1* mutation prevents the formation of all but one tooth (McGraw et al., 2011); however, given that this single tooth is of a size matching that expected for an adult zebrafish tooth (Wautier et al., 2001), it must have undergone multiple replacement cycles, indicating that replacement *per se* is not disturbed.

Our gain-of-function approaches yielded disjunct results in zebrafish, yet none of them indicating enhanced tooth development and/or replacement when constitutively activating the Wnt pathway. First, mutations in *axin1* do not have any visible effect on the tooth phenotype. Second, activating the pathway with LiCl had variable outcomes, either resulting in the absence, or the delayed formation, of teeth 3V^1^ and 5V^1^, or yielding a regular dentition with normal replacement, but no supernumerary teeth or accelerated tooth development or replacement. The occasional finding of an elaborately folded enamel organ (cf. Figure 5F) is discussed further. Given that LiCl and KCl treatment *in vitro* yielded results that were not very dissimilar, these data should not further be considered. Mutations in *apc* displayed a general developmental delay and early lethality, masking potential effects of this mutation on the dentition. Zebrafish heterozygous for this mutation have been reported to develop highly proliferative intestinal neoplasias (Haramis et al., 2006).

Thus, while the dynamic pattern of *dkk1* expression suggests an involvement of Wnt signaling at the time a replacement tooth is formed, both gain- and loss-of function approaches appear to work inhibitory (if at all) on tooth formation, but do not appear to affect tooth replacement. The seemingly similar phenotypes with gain-of-function (as with LiCl) or loss-of-function (as with *lef1* mutants, McGraw et al., 2011), or the lack of phenotype altogether when manipulating Wnt signaling (as in *mbl* mutants), is puzzling, especially in the light of the reported implication of Wnt signaling in the formation of ectodermal appendages, including teeth, in squamates and in mammals. Moreover, the results stand in sharp contrast with the strong tooth phenotypes generated when manipulating other signaling pathways in zebrafish. For example, ectopic expression of FGF ligands in zebrafish embryos results in supernumerary primary teeth (Jackman et al., 2013), while blocking fgf signaling results in arrest of primary tooth formation (Jackman et al., 2004). Below, we offer a number of explanations for our results, and point out some caveats when assessing the role of Wnt signaling in tooth replacement in zebrafish (and other polyphyodonts).

### Time window and dose-dependency

Because of the frequent occurrence of functional redundancy of pathway components, Wnt loss-of-function approaches are generally more difficult to achieve than gain-of-function approaches. We therefore focused primarily on stimulating the pathway. Two factors potentially explaining the puzzling and in some cases contradictory results are the time window in which Wnt signaling may be active, and dose-dependency. Jackman et al. (2013) manipulated fgf signaling between 12 and 20 hpf, i.e., more than 24 h before the first evidence of initiation of the first tooth, in order to have phenotypic effects on tooth formation. We treated at various time points, but mostly well-over 24 h before formation of the first replacement tooth. On the other hand, the occasional finding of an unusually folded enamel organ, displaying what looks like several tooth anlagen (cf. Figure 5E) and reminiscent of what was reported in mice with stabilized epithelial β-catenin (Figure 2B in Liu et al., 2008), may suggest a very precise and restricted time window where activation could produce supernumerary teeth. The variable results may furthermore be explained by the amount of LiCl that is actually physiologically interacting with the cells. We must also consider the possibility that Dkk and Wnt act through a reaction-diffusion mechanism, and that moderate and strong overexpression of the activator can have differential effects. For example, applying a computational model, Sick et al. (2006) show how levels of activator can affect patterning of hair follicles. Interestingly, Parsons et al. (2014) found both loss- and gain-of-function bone phenotypes in cichlid larvae treated with LiCl, and suggested that variation in Wnt levels can have a potent effect on craniofacial development.

### The complexity of the Wnt pathway

A second source of challenges to understand the role of Wnt signaling stems from the many proteins involved in the Wnt pathway, and their mutual interactions.

An overwhelming body of evidence supports the function of Dkk1 in Wnt/β-catenin signaling, but it has been suggested that Dkk1 and -2 may have also β-catenin-independent functions (Niehrs, 2006). A stimulatory effect has been reported for DKK, albeit known sofar for dkk2 only (Niehrs, 2006). Secreted Frizzled-related proteins sFRP1 and sFRP2, representing another type of Wnt antagonists (Kawano and Kypta, 2003), may act differently according to their physiological level: activating canonical Wnt signaling at low physiological level, inactivating the pathway with enhanced expression (Esteve et al., 2011).

Mutations in two proteins of the destruction complex, *AXIN-2* and *APC*, while both disabling β-catenin phosphorylation and promoting its nuclear localization, have completely opposite effects in humans: mutations in *AXIN-2* lead to hypodontia (Lammi et al., 2004), mutations in *APC* to hyperdontia (Thakker et al., 1995). This conflicting result may perhaps be explained by the type of mutation involved, its localization, the enormous size difference between both proteins, as well as how they function in the destruction complex (Roberts et al., 2011).

Functional redundancy of axins may explain the lack of a phenotype in the case of the *mbl* mutants, especially if *axin2* in zebrafish would turn out to have a more restricted expression domain than *axin1*, as is the case in the mouse. In the mouse, *Axin1* is uniformly expressed in the developing jaws, while *Axin2* is expressed in a more restricted pattern (Lammi et al., 2004). Interestingly, inactivation of β-catenin/Wnt signaling in zebrafish leads to loss of rostral taste buds, but leaves the caudal organs unaffected (Kapsimali et al., 2011). These authors did however not consider functional redundancy of axins as a possible explanation but attribute this observation to differential utilization of signaling pathways. Importantly, *Axin2* was identified as a direct Wnt target, providing a negative feedback loop (Jho et al., 2002).

LiCl, while commonly used as a Wnt activator, may not be very specific for just activating the Wnt pathway. Lithium ions also inhibit some non-kinase targets, such as inositol monophosphatase and histone deacetylase (Cohen and Goedert, 2004).

Moving down the signaling pathway, it is known that blocking the ubiquitination machinery is not sufficient to activate transcription of Wnt targets. The latter depends on the phosphorylation status of β-catenin, as only N-terminus dephosphorylated β-catenin is able to transduce the Wnt signal (Staal et al., 2002). Furthermore, nuclear localization of β-catenin may not be sufficient for gene transcription. Certain factors are known to antagonize transcriptional activity of Lef/Tcf or block the transcriptional potential of β-catenin. Baarsma et al. (2013) cite more than 30 of such proteins. Also, Lef1 can interact with the cofactor Aly to activate genes in the absence of Wnt signals (Bruhn et al., 1997). Finally, β-catenin/LEF1 signal activity is possibly achieved via a Wnt-independent mechanism, at least in hair follicle placodes (Huelsken et al., 2001).

These are just a few examples highlighting the complexity of Wnt signaling, and they suggest that simplifications of the pathway as is commonly done in many schematic representations, including the one in Figure 1, may be very misleading.

### Interspecific differences

Could Wnt signaling function in odontogenesis in distinctive ways in amniotes vs. non-amniotes, or in actinopterygians vs. sarcopterygians? Conservation of ancient signaling pathways, such as Wnt, across the animal kingdom, suggests that this is unlikely. Yet, to the least, certain proteins in the canonical Wnt signaling pathway function differently in zebrafish, in *Xenopus*, and in amniotes, e.g., (i) mutations in axin cause axis duplication in the mouse (Zeng et al., 1997), but reduced or absent eyes and telencephalon in zebrafish (Heisenberg et al., 2001; van de Water et al., 2001); (ii) in the mouse, eliminating Frat, a canonical Wnt pathway activator that binds to and inhibits GSK-3β after interacting with Disheveled (Dsh), has no effect (van Amerongen et al., 2005), while in *Xenopus* elimination of its homolog GBP prevents axis formation (Yost et al., 1998); (iii) there may be differences in switch mechanisms that control levels of and exchange between cytoplasmic and membrane associated pool of β-catenin (Brembeck et al., 2006); (iv) in mammalian cells, NLK (nemo-like kinase), when active, phosphorylates LEF-1/TCF and prevents β-catenin –LEF-1/TCF from binding to DNA and subsequent activation of transcription (Ishitani et al., 2003). In zebrafish, NLK is believed to act rather as a co-activator to derepress genes inhibited by LEF-1/TCF (Thorpe and Moon, 2004). In many cases, the cell, rather than the signal, determines the nature of the response to Wnt pathway activation (Logan and Nusse, 2004). Conversely, the nature of the Wnt signal may determine a stimulatory or inhibitory effect, as shown in the case of fin regeneration (Stoick-Cooper et al., 2007). Finally, it has become clear that the concept of individual and independent Wnt signaling pathways is no longer tenable; it is likely that Wnt proteins activate a complex intracellular signaling network rather than individual pathways (Kestler and Kühl, 2008).

Can a lower level of conservation of the tooth developmental programme explain differential results following Wnt activation between actinopterygians and crossopterygians? The gene *eve1* has been shown to be expressed during initiation of tooth formation in zebrafish, but it is not involved in tooth formation in the mouse (Laurenti et al., 2004). Given that *eve1* expression is completely abolished by 300 mM LiCl treatment for 30 min. (Joly et al., 1993), defective tooth formation in lithium treated zebrafish may result from a repression of *eve1* expression rather than from overstimulation of the Wnt pathway. Interestingly, LiCl also prevents formation of structures evolutionarily related to teeth, the lepidotrichia, in *Corydoras aeneus* (Zarnescu et al., 2013).

### Precise knowledge of the pattern is required for valid interpretations

To correctly assess a dentition phenotype, it is important to thoroughly know pattern, position, number, size, and shape of the teeth. In many polyphyodont species with unlimited growth, data may be confounded by intraspecific variation in tooth numbers, size-dependent addition of tooth loci, simultaneous presence of different tooth generations, etc. The zebrafish, with its fixed number of teeth, whose development and replacement start at well-known time points, offers an enormous advantage over other teleost species. For example, an attached tooth is not normally present as a single tooth in controls at 5 dpf (Figure 6E in Parsons et al., 2014) and thus the reported effect of LiCl in the above study needs to be reassessed. It is furthermore important to use imaging methods that allow unequivocal assessment of the presence of tooth germs prior to any matrix deposition. Finally, external phenotypes may not reveal the extent of an underlying developmental delay, as for example in the *apc* mutants studied here, and therefore lead to unjustified interpretations.

## Conclusion

In conclusion, our data on expression of the soluble Wnt inhibitor *dkk1* suggest that Wnt signaling is active in a time window between late cytodifferentiation of a tooth, and early morphogenesis of its successor, hinting at a role specific in tooth replacement. Gain-of function approaches, however, if of any effect at all, tend to prevent primary tooth formation but not replacement. Loss-of-function approaches likewise appear to affect the number of loci, but not replacement *per se* (McGraw et al., 2011). Our results, which also include the occasional unusual folding of the enamel organ, point to a potential importance of fine-tuning of dosage, and a role of Wnt signaling in tooth replacement that is at least far more complex than hitherto assumed. It is hoped that the above analysis can be inspiring to critically assess the role of Wnt signaling in tooth development in polyphyodonts.

### Conflict of interest statement

The authors declare that the research was conducted in the absence of any commercial or financial relationships that could be construed as a potential conflict of interest.
